# Omitting duodenal biopsy in children with suspected celiac disease and extra-intestinal symptoms

**DOI:** 10.1186/s13052-017-0377-5

**Published:** 2017-07-15

**Authors:** Mauro Bozzola, Cristina Meazza, Chiara Gertosio, Sara Pagani, Daniela Larizza, Valeria Calcaterra, Ombretta Luinetti, Giovanni Farello, Carmine Tinelli, Lorenzo Iughetti

**Affiliations:** 1 Department of Internal Medicine and Therapeutics, Pediatrics and Adolescentology Unit, University of Pavia, Fondazione IRCCS Policlinico San Matteo, Piazzale C. Golgi 19, 27100 Pavia, Italy; 2 Fondazione IRCCS Policlinico San Matteo, Pavia, Italy; 3 Department of Pathology, Fondazione IRCCS Policlinico San Matteo, Pavia, Italy; 4 Pediatric Unit, Department of Life Health and Environmental Sciences, University of L’Aquila, L’Aquila, Italy; 5 Clinical Epidemiology and Biometric Unit, Fondazione IRCCS Policlinico San Matteo, Pavia, Italy; 6 Department of Pediatrics, University of Modena and Reggio Emilia, Modena, Italy

**Keywords:** HLA genotype, Anti-transglutaminase antibodies, Anti-endomysial antibodies, Celiac disease, Children

## Abstract

**Background:**

The aim of our study is to evaluate if in children with highly positive serology and HLA-DQ2/DQ8 (triple test, TT) and only extra-intestinal symptoms, it is possible to omit performing an intestinal biopsy for celiac disease (CD) diagnosis, as suggested by the new European Society for Pediatric Gastroenterology, Hepatology and Nutrition ESPGHAN guidelines.

**Methods:**

In this retrospective study a total of 105 patients, suspected of having CD because of extra-intestinal symptoms and showing serum tissue transglutaminase antibody (anti-tTG) and anti-endomysial antibody (EMA) measurements and HLA genotyping, were considered for the final analysis (33 boys and 72 girls; age range 1.5–17.6 years).

**Results:**

Histological findings confirmed diagnosis of CD in 97 (92.4%) patients. Forty-one patients (39%) showed anti-tTG >10 times normal values, positive EMA and positive HLA-DQ2/DQ8 (positive TT). All of them had a diagnosis of CD, therefore there were no false positive cases. Sixty-four patients were negative for the TT. In eight cases, CD was ruled out and these were considered true negative cases. In the remaining 56 negative TT patients, intestinal biopsy confirmed CD diagnosis and they were considered false negatives. Based on these results, specificity for the TT was 100% and sensitivity was 42.3%.

**Conclusions:**

On the basis of the present study, diagnosis of CD can be reliably performed without a duodenal biopsy in children with only extra-intestinal symptoms.

## Background

According to the first guidelines established by the European Society for Pediatric Gastroenterology, Hepatology and Nutrition (ESPGHAN) in 1969 [1], celiac disease (CD) diagnosis depended on gluten-dependent symptoms and characteristic histological changes (villous atrophy, crypt hyperplasia, increase of intraepithelial lymphocytes) found in the duodenal biopsy.

Anti-endomysial antibodies (EMA) were discovered in the 1980s [2] and tissue transglutaminase antibodies (anti-tTG) were identified as a celiac autoantigen in the late 1990s [3]. Furthermore, in the last few years both the sensitivity and specificity of the serological tests have increased to nearly perfect values and some studies have been published suggesting that these tests alone may be sufficient to confirm the diagnosis of CD [4, 5]. In addition, a strong association of CD with the genetic markers human leukocyte antigen (HLA)-DQ2 and/or HLA-DQ8 has been established [6]. Therefore, considering these new scientific and technical developments and using an evidence-based approach, a working group within ESPGHAN formulated guidelines for CD diagnosis offering the option of not performing the intestinal biopsy in children and adolescents [7]. The requirements for omitting intestinal biopsy are gastrointestinal symptoms and signs suggestive of CD, anti-tTG antibody levels of more than 10 times the upper normal limit, positive confirmation of EMA and the presence of at risk HLA-DQ2 or HLA-DQ8. If all requirements are fulfilled, a gluten-free diet (GFD) is started and the patient is followed for improvement in symptoms and decrease of autoantibodies.

In the last few years, many studies have obviously been aimed at validating the indications of the ESPGHAN guidelines. Some of them confirmed that performing a duodenal biopsy can be omitted in a selected population of children and adolescents [8–10], while some others claimed that prudence is necessary in making a diagnosis of CD without duodenal biopsy [11, 12]. In fact, some authors suggest that symptoms are an essential part of CD diagnosis, although serological tests are highly sensitive and specific for CD [10]. A recent description of a child with a potential CD underlines the fact that strictly following the ESPGHAN guidelines could avoid intestinal biopsy but would consider a patient who is perhaps just potentially affected as celiac [13]. It is therefore important to repeat the CD serology before starting a GFD without a biopsy.

It is well known that CD can manifest with only extra-intestinal symptoms, the so-called non-classical CD, such as iron and folic acid deficiency with or without anaemia, dermatitis herpetiformis, delayed puberty, short stature, enamel defects, recurrent aphthous stomatitis, etc. [14].

The aim of our retrospective study was to evaluate whether serological markers combined with determination of HLA-DQ2/DQ8 genotype could replace the intestinal biopsy in children suspected of having CD but with only extra-intestinal symptoms.

## Methods

We conducted a retrospective study involving 268 children and adolescents suspected of having CD due to extra-intestinal symptoms (Table 1). They had been referred to various highly specialised Italian Endocrinological Centres: Unità di Pediatria Auxologia, Fondazione IRCCS Policlinico San Matteo, Pavia; Dipartimento di Pediatria, Università di Modena and Reggio Emilia, Modena and Ospedale de L’Aquila, L’Aquila. All research was performed according to the Ethical Standards involving human participants. The study was approved by the Ethics Committee “Comitato Etico Area di Pavia” of the Fondazione IRCCS Policlinico San Matteo. All participants provided parental written informed consent. Firstly, children were checked for anti-tTG and/or EMA. Then, as a consequence of positive serology, they underwent intestinal biopsy and in most cases HLA-DQ2/DQ8 genotyping was performed. The histology considered the standard criterion for CD diagnosis according to the 2012 ESPGHAN guidelines [7].

**Table 1:** Extra-intestinal symptoms of the enrolled patients suspected of CD

• Failure to thrive	
• Dhort stature	
• Delayed puberty	
• Weight loss	
• Iron deficiency anaemia	
• Enamel defects	
• Dermatitis herpetiformis	

According to the aim of our study and based on the ESPGHAN guidelines for the diagnosis of CD in children [7], we decided to consider anti-tTG, EMA and HLA-DQ2/DQ8 as a triple test (TT) for the diagnostic approach. We defined a TT as positive if anti-tTG was >10 times normal values, together with positive EMA and a positive HLA-DQ2 and/or DQ8. All three parameters had only been measured in 105 patients and these were thus considered for the final analysis. Thirty-three (31.4%) were boys and 72 (68.6%) were girls. The age range was 1.5–17.6 years, mean (SD) being 7.6 (4.2) years; only seven (6.7%) were younger than 2 years.

The other 163 children showed positive anti-tTG (although not always >10 times normal values) and/or positive EMA. Only in 31 out of 163 children had HLA genotyping been performed and showed a positive HLA-DQ2 and/or DQ8. One hundred fifty six children were diagnosed as celiac according to the result of intestinal biopsy, while the remaining seven children did not show histological findings suggestive of CD.

EMA were detected by indirect immunofluorescence on sections from the distal portion of monkey oesophagus, as an antigenic substrate, using a commercially available assay kit (GmbH Labordiagnostik, München). Serum anti-tTG IgA and IgG levels were determined by a commercially available enzyme-immunosorbent assay (ELISA) kit (Eu-tTG IgA and IgG, Eurospital, Trieste, Italy).

To evaluate the presence of celiac-susceptible DQ heterodimers, the patients were typed for HLA class II polymorphisms by PCR-SSP at high resolution level. DNA was extracted by a salting out procedure [15]. Polymorphism within the exons 2 of HLA-DQA1 and DQB1 genes was defined using a polymerase chain reaction with sequence specific primers (PCR-SSP) [16].

### Statistical analysis

Quantitative variables are described as mean and standard deviation (SD); qualitative ones are described as counts and percentages. Ninety five percent Confidence Intervals (CI) were calculated for sensitivity, specificity, positive and negative predictive value. Comparisons between the different groups were evaluated with Fisher’s exact test. The data analysis was performed with the STATA statistical package (release 14.0, 2015, Stata Corporation, College Station, Texas, USA).

## Results

Diagnosis of CD was done in 97 (92.4%) patients by the confirmation with histological findings on intestinal biopsy. After the diagnosis of CD was made, GFD was recommended to all patients.

**Fig. 1:**
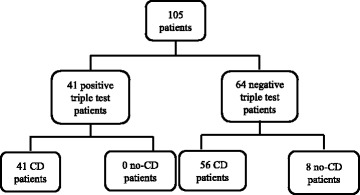
Results of the triple test in our population. CD = celiac disease

Only 41 patients (39%) were positive for the triple test (Fig. 1). All of them had either Marsh 2 or Marsh 3 histological lesions and a definitive diagnosis of CD was made. These were true positive cases for the TT. Thus, the positive predicted value (PPV) of the TT was 100% (95% CI: 96%–100%) (Table 2). There were Therefore no false positive cases.

**Table 2:** Efficiency of the triple test (TT) in patients with extra-intestinal symptoms, suspected of having CD

	Positive biopsy *n* = 97	Negative biopsy *n* = 8	
Positive TT (*n* = 41)	True positive 41	False positive 0	Positive predictive value 100%
Negative TT (*n* = 64)	False negative 56	True negative 8	Negative predictive value 12.5%
	Sensitivity 42.3%	Specificity 100%	

Sixty-four patients were negative for the triple test. In eight cases, CD was ruled out by duodenal biopsy and these were considered true negative cases (Fig. 1, Table 2). Five of eight had normal histology, two had Marsh 1 histological lesions and were classified as potential CD and one had a diagnosis of duodenopathy. All subjects with negative TT were HLA-DQ2/DQ8 positive but had either positive anti-tTG or EMA. Only one girl showed both negative EMA and IgA-tTG. In the remaining 56 patients with negative TT, intestinal biopsy confirmed the diagnosis of CD and they were considered false negatives. Based on these results, specificity for the TT was 100% (95% CI: 96%–100%) and sensitivity was 42.3%, (95% CI: 33.2%–52.9%), while negative predictive value (NPP) was 12.5% (95% CI: 6.7%-20.2) (Table 2).

**Fig. 2:**
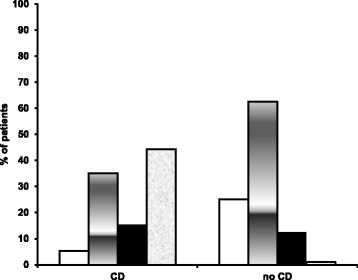
Distribution of patients by anti-tTG levels. Negative anti-tTG (*white bar*); 1–5 times normal values (*grey bar*); 6–10 times normal values (*black bar*); >10 times normal values (*shaded bar*)

In the CD group, 44.3% of patients showed anti-tTG levels >10 times normal values, 15.4% 6–10 times normal values, 35.1% 1–5 times normal values and 5.2% negative values (Fig. 2). On the contrary, the percentages of patients according to anti-tTG values were different in the no-CD group: no patients showed anti-tTG levels >10 times normal values, 12.5% 6–10 times normal values, 62.5% 1–5 times normal values and 25% negative values (Fig. 2) (*p* < 0.001).

## Discussion

Our retrospective study tested the performance of the ESPGHAN guidelines for the non-biopsy CD diagnosis in children with only extra-intestinal symptoms.

For decades, the diagnosis of CD has been based on the histological analysis of duodenal biopsy samples, preferably taken during an upper endoscopy [17]. However, growing evidence indicates the pitfalls of histology due to the number, site, size and orientation of biopsy samples [18]. Serological testing of the so-called celiac antibodies has become increasingly important for the diagnosis of CD. In particular, the discovery of the enzyme tTG as the major autoantigen involved in CD pathogenesis led to the development of routine assays for anti-tTG determination [19]. Furthermore, HLA genotyping results, with a high diagnostic sensitivity but a low specificity, have a primary role in excluding CD [20]. Taking into account all these factors, there is a growing interest in the debate over the omission of performing duodenal biopsies according to the ESPGHAN guidelines for the diagnosis of CD in children and adolescents in the presence of symptoms and high levels of anti-tTG [5]. More recently, the British Society of Paediatric Gastroenterology, Hepatology and Nutrition (BSPGHAN) also reviewed its diagnostic guidelines for CD, reaching similar conclusions [21]. A very recent prospective study showed that diagnosis of CD can be reliably performed without a duodenal biopsy in approximately 11% of cases, selected applying the ESPGHAN/BSPGHAN guidelines in a restricted way, considering as symptomatic only those cases with diarrhoea, weight loss, failure to thrive and iron deficiency anaemia [22]. However, some studies suggest prudence in omitting performance of a duodenal biopsy in these children. Guandalini and colleagues expressed concerns about the skipping of duodenal biopsy to confirm the diagnosis of CD. They demonstrated that if the diagnosis of CD is made without the endoscopy, any unexpected disorders of the upper gastrointestinal tract will be missed. They therefore suggest that it is mandatory to closely follow up patients on a GFD and evaluate those with unresolved symptoms [11].

In our study, only 39% of patients suspected for CD showed positivity for the TT and would have been eligible for diagnosis without intestinal biopsy, according to the ESPGHAN guidelines. We found that all the TT positive patients were celiac and the specificity for this test was 100%, confirming that this test is reliable and that it is possible to not perform a duodenal biopsy also in patients with only extra-intestinal symptoms. However, a prudent approach is suggested when a diagnosis of CD is made without intestinal biopsy, since the anti-tTG antibody titre could sometimes be elevated due to a gastrointestinal infection and not to CD, as shown in the case reported by Schirru et al. [12]. More recently, another case report described a two-year-five-month-old child with familiarity for CD, elevated anti-tTG antibodies and positive EMA who was negative to biopsy and classified as potential CD [13]. Therefore, it is important to closely follow up this kind of patient for some weeks and, if necessary, repeat the serology before starting a GFD without a biopsy. Otherwise, a patient who is only potentially affected or has elevated anti-tTG levels due to other causes would be considered as celiac.

On the contrary, we found that the sensitivity of TT was only 42.3%, suggesting that biopsy is necessary for confirming the diagnosis in patients with negative TT, even if the anti-tTG titre is high and they show HLA-DQ2 or -DQ8. In fact, 56 out of 64 patients suspected of having CD but with negative TT were celiac subjects and only eight did not show the disease. Furthermore, three of the latter showed histological lesions due to different duodenopathies.

Triple test should therefore not be used as a screening test for CD, because of its low sensitivity, much lower than anti-tTG measurement sensitivity. Therefore, when clinicians are faced with patients in whom CD symptoms are only extra-intestinal and serological markers are not so elevated, intestinal biopsy is mandatory in order to avoid the risk of these patients remaining undiagnosed and being exposed to the risk of long-term complications, such as osteoporosis, infertility and cancer, especially lymphomas [23]. Recently, our group reported the development of positive serology for CD in a child without gastrointestinal symptoms and only growth retardation, who had previously been investigated for CD and proved negative [24], further suggesting that a close follow-up of patients in whom a CD diagnosis is difficult is very important.

## Conclusion

On the basis of the present study, diagnosis of CD can be reliably performed without a duodenal biopsy also in children with only extra-intestinal symptoms and the characteristics suggested by the ESPGHAN guidelines. However, these patients represent only a small part of all patients with only extra-intestinal symptoms investigated for CD. In the other group of subjects, duodenal biopsy remains mandatory to confirm CD diagnosis.

## Abbreviations

Anti-tTG: Tissue transglutaminase antibodies
BSPGHAN: British Society of Paediatric Gastroenterology, Hepatology and Nutrition
CD: Celiac disease
CI: Confidence intervals
ELISA: Available enzyme-immunosorbent assay
EMA: Anti-endomysial antibodies
ESPGHAN: European Society for Pediatric Gastroenterology, Hepatology and Nutrition
GFD: Gluten-free diet
HLA: Human leukocyte antigen
NPP: Negative predicted value
PPV: Positive predicted value
SD: Standard deviation
TT: Triple test
